# Oral verruciform xanthoma and erythroplakia associated with chronic graft-versus-host disease: a rare case report and review of the literature

**DOI:** 10.1186/s13104-017-2952-7

**Published:** 2017-11-28

**Authors:** Giorgia Capocasale, Vera Panzarella, Pietro Tozzo, Rodolfo Mauceri, Vito Rodolico, Dorina Lauritano, Giuseppina Campisi

**Affiliations:** 1 Department of Surgical, Oncological and Oral Sciences, University of Palermo, Via del Vespro 129, 90127 Palermo, Italy; 2 Human Pathology, Department of Health Promotion & Mother and Child Care, University of Palermo, Via Alfonso Giordano 3, 90127 Palermo, Italy; 3 Department of Medicine and Surgery, University of Milan-Bicocca, Via Cadore, 48, 20900 Monza, Italy

**Keywords:** Chronic graft versus-host-disease, Verruciform xanthoma, Oral potential malignant disorder, Erythroplakia

## Abstract

**Background:**

Oral verruciform xanthoma is an uncommon benign lesion. Although oral verruciform xanthoma occurs in healthy individuals, it has been also reported in association with some inflammatory conditions. The aim of this study is to report a case of oral verruciform xanthoma associated with chronic graft-versus-host disease and to review the literature on this topic.

**Case presentation:**

A 47-year-old Caucasian male presented to the Sector of Oral Medicine “V. Margiotta”, University Policlinic “P. Giaccone” of Palermo complaining of a mass on the gingiva. He first noticed the painless mass 1 year ago. He reported to have undergone allogenic hematopoietic stem cell transplantation 15 years ago for acute lymphoblastic leukaemia. Intraoral examination revealed a well-circumscribed, sessile yellowish and verrucous nodule upon canine, multiple yellowish and verrucous nodules on the hard palate, yellowish and verrucous nodules on left buccal mucosa. In addiction an area of white striae in a reticular pattern with erythema and ulceration was present on the dorsum of the tongue. This lesion was consistent with a known history of oral chronic graft versus host disease. Moreover, we observed a suspected area of oral erythroplakia yet on the dorsum of the tongue. In biopsy specimen of hard palate histopathological examination revealed a diagnosis of verrucous xanthoma of the oral cavity; in addiction in biopsy specimen of the dorsum of the tongue revealed the presence of erythroplakia with high grade dysplasia.

**Conclusion:**

Verruciform xanthoma of the oral cavity associated with chronic graft-versus-host disease is a rare condition with a usually benign clinical course but malignant transformation has been described in association with oral potential malignant disorder (e.g. chronic graft versus host disease, erythroplakia). Very rare cases showed association with oral chronic graft versus-host-disease. To date, only eight cases were published in the world literature. Therefore it could be important follow up patients also for oral verruciform xanthoma onset.

## Background

Oral verruciform xanthoma (OVX) is rare, benign unique lesion [1]. This lesion grows slowly, usually is asymptomatic, and clinically is similar to others epithelial hyperplasias such as verruciform plaques and papule. Shafer, in 1971, described for the first time 15 cases of OVX in the mucosa [2]. The frequency ranges from 0.025 to 0.094%, most in adults without predilection of sex [1]. The occurrence of multiple or disseminated OVX is extremely rare [3].

This unique oral lesion usually appears on masticatory gingival mucosa, but it may also manifests on hard palate and tongue, and on non-keratinized mucosa such as, buccal mucosa, and floor of the mouth, alveolar mucosa, and soft palate [4]. These hyperplastic lesions are yellowish, white, pink or grey [1]. OVX colour differences are dependent on the thickness of the epithelium overlying. Differential diagnosis includes squamous papilloma, verruca vulgaris, verrucous carcinoma and squamous cell carcinoma [4]. Thus, histopathological examination is essential for the differential diagnosis.

Three different types of OVX have been described according to clinical aspect and microscopic features [5]. The verrucous OVX, histologically shows hyperparakeratosis, acanthosis and elongation of the rete ridges. The papillary OVX differs from the others for stratified squamous epithelium, which appears as fingerlike elongations, filled with connective tissue. The flat OVX exhibits mild acanthosis, variable elongation of rete ridges and mild parakeratosis. The flat OVX is the most common type [5].

The pathogenesis of OVX is unknown; in fact, even if in this lesions foam cells containing lipids are increased, hyperlipidaemia in the submucosa or dermis has not been detected. One study suggested that periodontal disease, mechanical trauma, tobacco smoking, alcohol consumption, drugs, or the use of partial or total dentures may promote OVX formation, but no local or systemic causes have been demonstrated [4]. Human papilloma virus (HPV) has not been demonstrated in OVX lesions and viral particles have not been identified ultrastructurally [5].

The pathogenesis of the OVX is thought to be a result of tissue damage, especially to the epithelium, which results in breakdown of the phospholipid-rich cell membranes. The lipids, released and then taken up by the cells in the vicinity, produce the lipid-laden cells seen by histopathology exam [5].

OVX usually manifests in healthy individuals, but an association with autoimmune diseases such as lichen planus, pemphigus vulgaris, discoid lupus and psoriasis has been well documented [6–8]. OVX has been described in association with in situ carcinoma and squamous cell carcinoma also [9]. In addiction multifocal verruciform xanthoma of higher aero-digestive tract have been reported in patients with disorders of lipid storage.

An association of OVX with oral chronic graft versus-host-disease (cGVHD) after allogenic hematopoietic stem cell transplantation (aHSCT,) has also been previously reported although in very few cases [10–13].

cGVHD is an immunoregulatory disorder which occurs after allogeneic hematopoietic-cell transplantation (aHCT) and often shares features of autoimmunity and immunodeficiency characterized by lesions clinically (reticular/hyperkeratotic striations and plaques, erythema, and ulcerations) and histologically (apoptosis of keratinocytes and destruction of the basal cells) similar to oral lichen planus [14]. Therefore in patients who have undergone bone marrow transplantation and develop oral cGVHD, is frequently identified microscopically destruction of the basal keratinocytes of the epithelium, and it is possible that basal keratinocytes chronic damage leads to occurrence of OVX [10].

The current treatment of OVX consists in surgical resection of the lesion. Recurrence and malignant transformation of this lesion are rarely documented, and usually OVX has a good prognosis [4].

The aim of this study is to describe of a case of OVX and erythroplakia simultaneously present in the oral cavity of the same patient, associated with cGVHD following aHSCT, and review of the literature.

## Case presentation

A 47-year-old Caucasian male presented to the sector of oral medicine “V. Margiotta”, University Policlinic “P. Giaccone” of Palermo complaining of a mass on the gingiva. He first noticed the painless mass 1 year ago. He reported to have undergone HSCT 15 years ago for acute lymphoblastic leukaemia and had developed cGVHD of the mouth, liver and lungs 24 months after transplantation. cGVHD compatible lesions were treated with dexamethasone 0.01% mouthrinse 5–10 ml as a mouthwash twice daily for 5 or 10 min for 1 month.

**Fig. 1:**
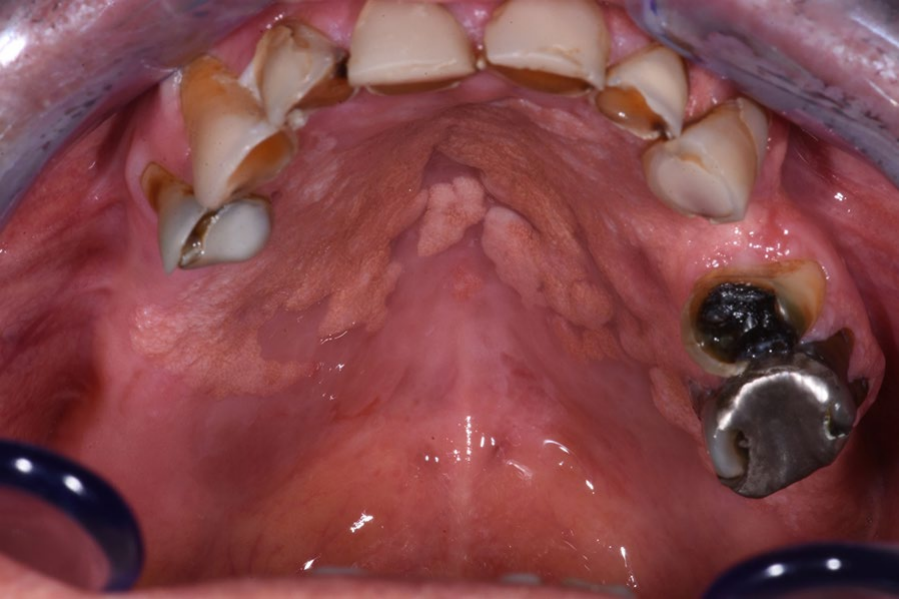
Well-circumscribed, sessile yellowish and verrucous nodule upon canine, multiple yellowish and verrucous nodules on the hard palate, yellowish

**Fig. 2:**
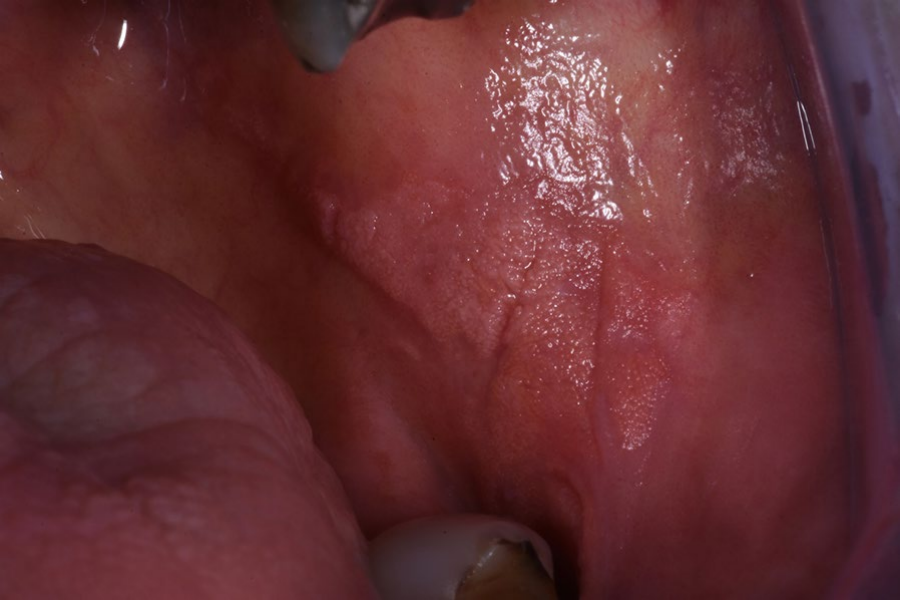
Verrucous nodules on left buccal mucosa

**Fig. 3:**
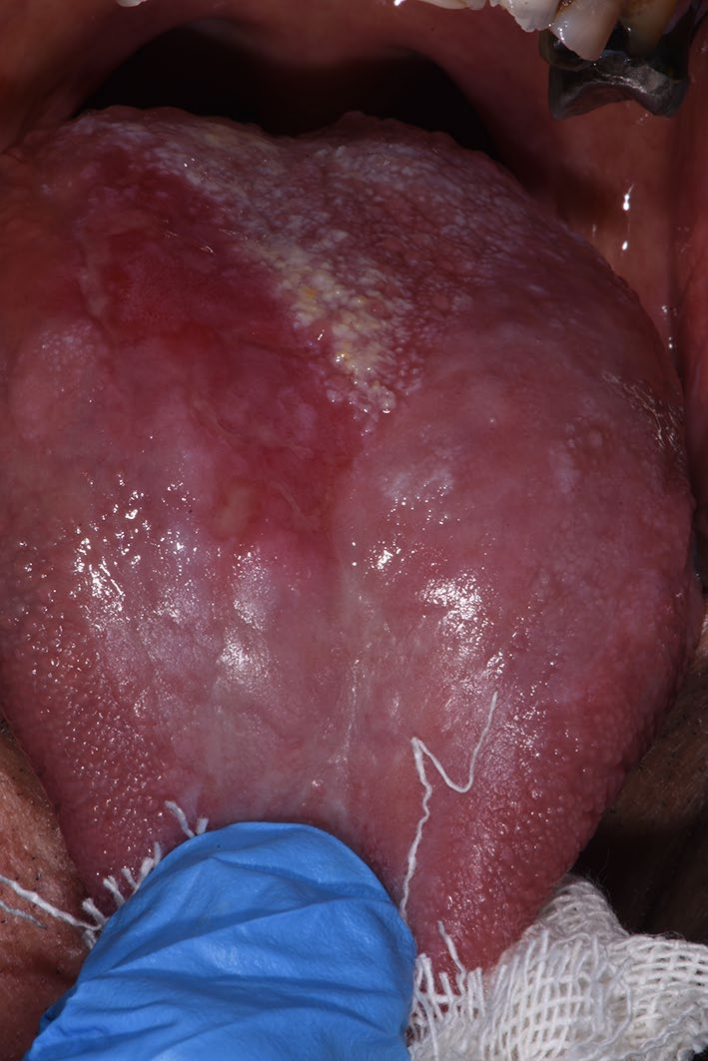
Area with white striae in a reticular pattern with erythema and ulceration on the dorsum of the tongue

Intraoral examination revealed a well-circumscribed, sessile yellowish and verrucous nodule upon canine, multiple yellowish and verrucous nodules on the hard palate (Fig. 1), yellowish and verrucous nodules on left buccal mucosa (Fig. 2). In addiction, an area of white striae in a reticular pattern with erythema and ulceration was present on the dorsum of the tongue. This lesion was consistent with a known history of oral cGVHD (Fig. 3). Moreover, we observed a suspected area of oral erythroplakia yet on the dorsum of the tongue.

After obtaining an informed consent from the patient, an incisional biopsy was performed with 6 mm diameter punches biopsy one in hard palate, and one on the dorsum of the tongue. The biopsy specimens were processed routinely in 10% formalin and embedded in paraffin.

**Fig. 4:**
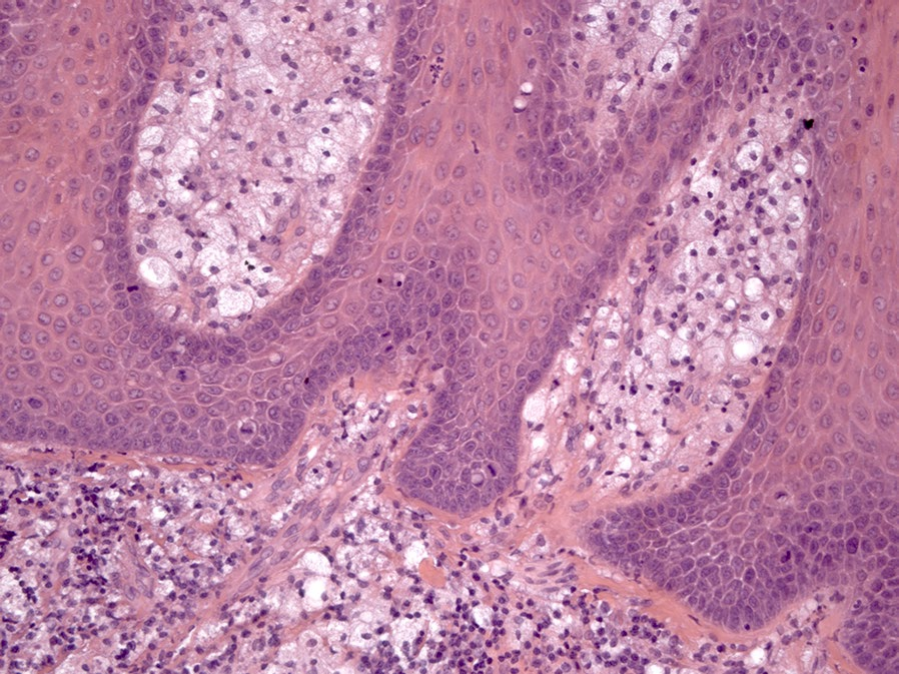
Connective tissue papillae are composed almost exclusively of large macrophages with foamy cytoplasm, also known as xanthoma cells (Hematoxylin & Eosin, ×200)

In biopsy specimen of hard palate histopathological examination revealed crypts between the epithelial projections filled with parakeratin, and rete ridges elongated to a uniform depth; connective tissue papillae composed of numerous large macrophages with foamy cytoplasm (Fig. 4). Based on these findings, a diagnosis of OVX was established.

**Fig. 5:**
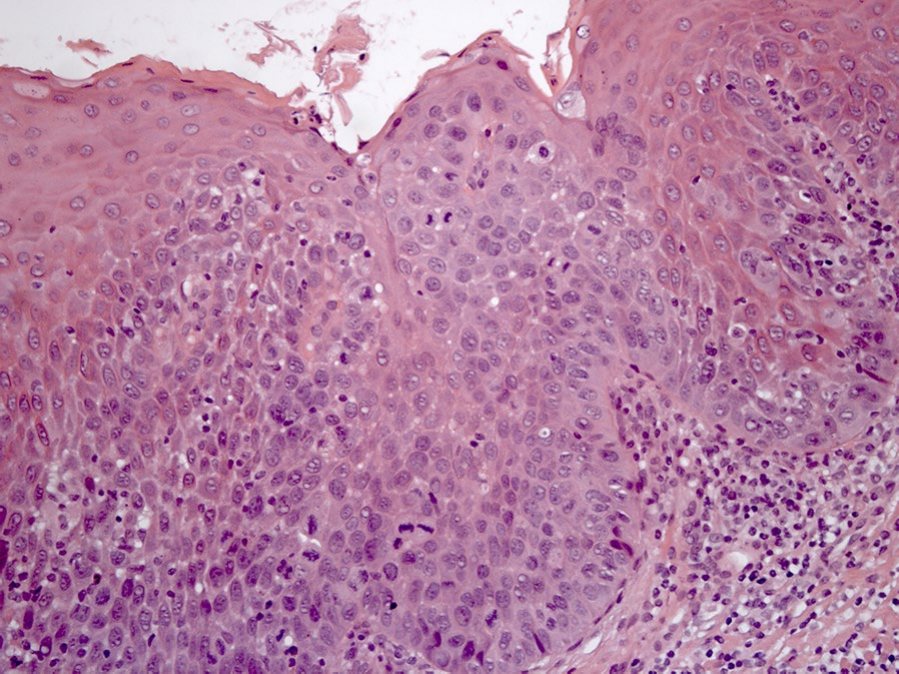
Cellular crowding and disordered arrangement are noted throughout most the epithelial thickness; the underlying connective tissue demonstrates chronic inflammation (Hematoxylin & Eosin, ×200)

In biopsy specimen of the dorsum of the tongue histopathological examination revealed epithelium with a lack of keratin production and nuclear hyperchromatism, pleomorphism and cellular crowding alterations from the basal layer throughout most the epithelial thickness, although slight maturation and flattening of the cells appeared to be present at the surface; the underlying connective tissue demonstrated chronic inflammation (Fig. 5). Based on these clinical-pathological findings, a diagnosis of erythroplakia with high grade dysplasia was established.

As previously reported, the current treatment OVX consists in surgical resection of the lesion, and recurrence and malignant transformation of this lesion are rarely documented, and usually OVX has a good prognosis [4].

## Discussion and conclusion

In this paper we have described a rare case of OVX associated with erythroplakia with high grade dysplasia in a patient who developed cGVHD following aHSCT. OVX may be misdiagnosed clinically as a malignant disorder viral warts or benign/premalignant mucosal disease. The present case illustrates the importance of clinico-pathological examinations for the diagnosis of OVX.

Since OVX is a rare formation, the simultaneous presence of erythroplakia makes this case report very unsual. cGVHD following aHSCT is the common denominator of the two lesions probably contributing to the development of both.

In fact, since the etiopathogenesis of OVX is unknown, it may be related to inflammation and tissue damage. Immunologic and microbiological factors have been also supposed [15]. Zegarelli et al. [16] proposed that, in general, verruciform xanthoma is caused by epithelial entrapment along the deep crypts, with subsequent degeneration and lipid formation. The lipids, in turn, are ingested by macrophages resulting in the formation of xanthoma cells. In the lamina propria, chronic inflammatory infiltration may also be observed [17].

However in patients who have undergone bone marrow transplantation and develop oral cGVHD, is frequently identified microscopically destruction of the basal keratinocytes of the epithelium and it is possible that basal keratinocytes chronic damage leads to occurrence of OVX [10]. In these cases, making differential diagnosis with malignant lesions is crucial.

In fact, major late complications of aHSCT are secondary malignancies including oral cancer and oral cGVHD-related inflammation [18]. The majority of the patients with oral cancer post-HSCT had a history of cGVHD. The recurrence of oral cancer is prevalent in patient post-HSCT, and usually manifests with an aggressive nature. This aggressive behaviour should be related by a unique genomic instability (GI) induced by chronic inflammation [19]. The unique genomic instability may led to oral cancer because the specific type of GI (tetranucleotide microsatellite instability) observed was frequently detected in sporadic carcinomas [20, 21]. So, patients with oral cancer post-HSCT, should be carefully monitored for recurrence of oral cancer with biopsies, when it is suspected.

Few authors have reported cases of OVX in patients with cGVHD [10–13]. Only eight cases were reported and all less extended of this. These cases are reported in Table 1. To our knowledge, our case report is a rare case of OVX and erythroplakia with high grade of dysplasia, simultaneously present in the oral cavity of the same patient, associated with cGVHD following aHSCT.

**Table 1:** Reported cases of oral verruciform xanthoma associated with oral chronic GVHD (cGVHD)

Author (year)	Country	N. of cases	Age/sex	site
Yu et al. (2007) [15]	Formosa	1	Not known	Not known
Allen et al. (1993) [10]	USA	1	22/M	Labial mucosa
Sibaud et al. (2006) [11]	France	1	57/M	Gingiva
Shahrabi Farahani et al. (2011) [12]	USA	5	45/M	Buccal mucosa
			13/F	Tongue
			63/M	Tongue
			43/F	Labial mucosa
			45/M	Hard palate
	Italy	1	47/M	Gingiva
				Hard palate
				Buccal mucosa

Here we report a case of OVX developed in association with oral cGVHD, bringing the total number of cases in the literature to nine (Table 1). Since oral cGVHD and oral lichen planus usually present in the buccal mucosa, tongue, this reinforces the idea that the pathogenesis of OVX is related to immunologic disorder. The trauma to the basal cell and apoptosis characteristic of lichen planus and oral cGVHD releases lipids phagocytosed by histiocytes or dermal dendrocytes. Other associated conditions such as pemphigus vulgaris and epidermolysis bullosa suggest a role for epithelial damage.

OVX is a rare condition with a usually benign clinical course, but malignant transformation has been described. The patients affected by OVX associated with cGVHD should be monitored regularly for the increased risk for mucosal carcinoma.

Previous therapies for aHSCT treatment (immunosuppressive drugs) increase the risk of developing squamous cell carcinoma of the mouth. cGVHD may be associated with OVX. This lesion must be distinguished from malignancy with a biopsy. In fact OVX requires biopsy for a definitive diagnosis, and a period follow-up without further intervention.

OVX is a rare lesion, so that dentist and oral and maxillofacial surgeons recognize this disease with difficult. Therefore, considering that there are limited numbers of articles, the publication of case reports improves the knowledge of this benign tumour and increases the diagnostic skills.

## Abbreviations

OVX: oral verruciform xanthoma
cGVHD: chronic graft versus-host-disease
aHSCT: allogenic hematopoietic stem cell transplantation
HCT: hematopoietic-cell transplantation
